# Retinal degeneration increases susceptibility to myopia in mice

**Published:** 2013-09-28

**Authors:** Hanna Park, Christopher C. Tan, Amanda Faulkner, Seema B. Jabbar, Gregor Schmid, Jane Abey, P. Michael Iuvone, Machelle T. Pardue

**Affiliations:** 1 Department of Ophthalmology, Emory University School of Medicine, Atlanta, GA; 2 Department of Pharmacology, Emory University School of Medicine, Atlanta, GA; 3 Rehab Center of Excellence, Atlanta VA Medical Center, Atlanta, GA

## Abstract

**Purpose:**

Retinal diseases are often associated with refractive errors, suggesting the importance of normal retinal signaling during emmetropization. For instance, retinitis pigmentosa, a disease characterized by severe photoreceptor degeneration, is associated with myopia; however, the underlying link between these conditions is not known. This study examines the influence of photoreceptor degeneration on refractive development by testing two mouse models of retinitis pigmentosa under normal and form deprivation visual conditions. Dopamine, a potential stop signal for refractive eye growth, was assessed as a potential underlying mechanism.

**Methods:**

Refractive eye growth in mice that were homozygous for a mutation in *Pde6b*, *Pde6b^rd1/rd1^ (rd1)*, or *Pde6b^rd10/rd10^* (*rd10*) was measured weekly from 4 to 12 weeks of age and compared to age-matched wild-type (WT) mice. Refractive error was measured using an eccentric infrared photorefractor, and axial length was measured with partial coherence interferometry or spectral domain ocular coherence tomography. A cohort of mice received head-mounted diffuser goggles to induce form deprivation from 4 to 6 weeks of age. Dopamine and 3,4-dihydroxyphenylacetic acid (DOPAC) levels were measured with high-performance liquid chromatography in each strain after exposure to normal or form deprivation conditions.

**Results:**

The *rd1* and *rd10* mice had significantly greater hyperopia relative to the WT controls throughout normal development; however, axial length became significantly longer only in WT mice starting at 7 weeks of age. After 2 weeks of form deprivation, the *rd1* and *rd10* mice demonstrated a faster and larger myopic shift (−6.14±0.62 and −7.38±1.46 diopter, respectively) compared to the WT mice (−2.41±0.47 diopter). Under normal visual conditions, the DOPAC levels and DOPAC/dopamine ratios, a measure of dopamine turnover, were significantly lower in the *rd1* and *rd10* mice compared to the WT mice, while the dopamine levels were similar or higher than WT in the *rd10* mice. Lower basal levels of DOPAC were highly correlated with increasing myopic shifts.

**Conclusions:**

Refractive development under normal visual conditions was disrupted toward greater hyperopia from 4 to 12 weeks of age in these photoreceptor degeneration models, despite significantly lower DOPAC levels. However, the retinal degeneration models with low basal levels of DOPAC had increased susceptibility to form deprivation myopia. These results indicate that photoreceptor degeneration may alter dopamine metabolism, leading to increased susceptibility to myopia with an environmental visual challenge.

## Introduction

Refractive development describes a critical period of early post-natal development in which visual input influences the match between eye growth and optical power. Emmetropia or zero refractive error describes a perfect match of optical power and length of the eye such that the image is focused on the retina. A mismatch of power and axial length produces either myopia, where the visual image forms in front of the retina, or hyperopia, where the visual image forms behind the retina. Abnormal refractive development most commonly results in myopia. The prevalence of myopia continues to rise, reaching 42% in the US [1] and as high as 96% in Asian countries [2]. The reason(s) for the increased prevalence of myopia are unknown, although genetic and environmental factors have been shown to influence refractive development [3-6].

Several systemic and ocular diseases have been associated with refractive errors (reviewed in [7]). Albinism, Cohen syndrome, Down syndrome, and Marfan syndrome are all associated with high myopia [7]. Furthermore, myopia is often associated with ocular abnormalities, such as cataracts, glaucoma, chorioretinal abnormalities, and optic disc abnormalities [8]. Refractive errors are often much more variable in patients with retinal diseases (reviewed in [9]). In the case of patients with the complete form of congenital stationary night blindness, refractions are highly myopic [10]. Patients with congenital stationary night blindness have a mutation in *NYX* that disrupts visual transmission between rods and ON bipolar cells. A mouse sharing the same mutation was more susceptible to form deprivation myopia [11]. Infants with retinopathy of prematurity have a paradoxical association of decreased eye growth and myopic refractions; this effect is also found in the retinopathy of prematurity rat model [12].

Patients with retinal degeneration also have characteristic refractive errors. For instance, hyperopia correlates with increased risk of age-related macular degeneration [13-15] and is found in patients with Leber congenital amaurosis [9]. Alternatively, cone-rod dystrophy has been associated with myopia [16,17]. The incidence of myopic refractive errors in patients with retinitis pigmentosa (RP) is 75%, while patients with the X-linked form of RP reach 95% [18]. The underlying cause of these associations is not known. Genetically altered mice may offer a tool for examining the mechanistic link between retinal degeneration and refractive development.

*Pde6b^rd1/rd1^ (rd1)* and *Pde6b^rd10/rd10^* (*rd10*) mice are two mouse models frequently used to study mechanisms of photoreceptor degeneration since these models share mutations with patients with RP [19,20]. These two models have a mutation in the *Pde6b* gene that encodes the β-subunit of cyclic nucleotide phosophodiesterase-6, with *rd1* mice having a non-sense mutation in exon 7 of *Pde6b* [21,22] and *rd10* mice with a missense point mutation in exon 13 of *Pde6b* [23]. The *rd1* mice were the first photoreceptor degeneration model to be described [24,25] and are characterized by fast rod degeneration followed by cone degeneration. In the *rd1* mice, the rods never fully develop before the onset of degeneration at postnatal day 8 (P8). Rod degeneration is complete by P20 with cones still viable past 18 months of age [26-29]. The *rd10* mice were first described in 2002 [30] and have a slower rate of rod degeneration, which starts at P16 with rods eliminated by P60 [23,31,32] and cones degenerating more slowly and still present past 9 months of age [31]. Interestingly, dopamine, a proposed “stop signal” in refractive development [33], has been reported to decrease in retinal degeneration [34-37]. The depressed level of dopamine in these degenerative models provides a potential mechanism for increased association/prevalence of myopia in human patients with retinal degenerative disorders.

In these studies, we examined whether retinal degeneration influences refractive development under normal visual conditions and alters susceptibility to form deprivation myopia. Experiments were performed starting at 4 weeks of age when rod photoreceptors were degenerated in the *rd1* mice [26-28] or at the peak of apoptosis in the *rd10* mice [31]. We correlated the levels of steady-state dopamine and its metabolite 3,4-dihydroxyphenylacetic acid (DOPAC) with the changes in eye size during unaltered or form deprivation visual conditions to further delineate whether dopamine activity or some other aspect of retinal degeneration affected visually driven eye growth.

## Methods

### Animals and experimental design

Animals used in this study were obtained from a breeding colony located at Atlanta Veterans Affairs Medical Center (VAMC). The colony was initiated with the following mice from the Jackson Laboratory (Bar Harbor, ME): wild-type C57BL/6J (WT; strain #000664), B6.C3-Pde6b^rd1^ Hps4^le^/J (*rd1*; strain #000002), and B6.CXB1-*Pde6b^rd10^*/J (*rd10*; strain #004297). No b-wave (*nob*) mice with a mutation in *Nyx* were obtained from an in-house breeding colony. All mice were housed in typical shoe-box cages (7.25 in wide × 11.5 in deep × 5 in high) with chow (Harlan 2018S Teklad Global 18% Protein Rodent Diet, Indianapolis, IN) and water accessible ad libitum. The room was kept under a 12 h:12 h light-dark cycle (12–25 lux). All experiments were approved by the Institutional Animal Care and Use Committee at VAMC and adhered to the Association for Research in Vision and Ophthalmology (ARVO) statement for the Use of Animals in Ophthalmic and Vision Research.

Refractive development of each strain was established by raising mice in unaltered visual conditions and measuring refractive error (WT, n=29; *rd1*, n=17; *rd10*, n=13) and axial length (WT, n=11; *rd1*, n=11; *rd10*, n=10) weekly from 4 to 12 weeks of age. To assess the effect of visual disruption in combination with the loss of photoreceptors, each strain underwent form deprivation (FD) with diffuser goggles. At 4 weeks of age, baseline measurements were recorded, and the mice immediately received a head-mounted diffuser goggle over the right eye, as described previously [38]. Briefly, after anesthesia (ketamine 80 mg/kg; xylazine 16 mg/kg), the skull was exposed, and three screws (4 mm × 0.7 mm) were placed around the intersection of the lambda and sagittal sutures. Acrylic was positioned around the screws and metal tubing that accepts the goggling frame. The goggle consisted of a contact lens uniformly coated with white nail polish. The mice were goggled for either 2 or 5 (WT only) weeks and assessed weekly for refractive error (WT: n=28 goggled, n=21 naïve controls; *rd1*: n=25 goggled, n=17 naïve controls; *rd10*: n=14 goggled, n=14 naïve controls) and axial length (WT: n=17 goggled, n=13 naïve controls; *rd1*: n=13 goggled, n=11 naïve controls; *rd10*: n=14 goggled, n=14 naïve controls). Experimental treatment groups consisted of 4 to 10 independent litters from each strain with no significant differences between litters.

### Refractive error

Refractive error of each mouse was assessed with an eccentric infrared photorefractor customized for the mouse eye by Dr. Frank Schaeffel [39,40]. The pupils were dilated with 1% tropicamide to ensure pupil sizes greater than 1.7 mm [39]. All measurements were done in a dark environment to maximize the detection of light reflected from the mouse pupil. The mouse was placed on a platform 60 cm from the photorefractor, and refractive error values were recorded with a custom software program (Mouse Refract, Version Aug.21, 2009, STZ Biomedizinsche Optik und Funktiosprunfung, Tübingen, Germany). As previously described, refractions were first measured in awake, gently restrained mice to gauge the range of refractions with natural head position [38], followed by sedated refractions (ketamine 80 mg/kg; xylazine 16 mg/kg). For each eye, an average of 50–200 measurements taken under sedation was used for analysis. In some instances, refractive values could not be obtained after sedation due to abnormal pupil reflections (i.e., irregular tear-film), and awake mouse recording values were used.

### Axial length measurements

Axial length measurements were obtained using two methods: partial coherence interferometry (PCI) and 1310 nm spectral domain-optical coherence tomography (SD-OCT). Following the awake measurements of refractive error and before the sedated refractions, axial length was measured with a custom-built PCI modified for the mouse eye [41]. Mice were placed in a restraining tube with their head secured by a clamp that attached to a head pedestal [41]. Thus, all mice undergoing PCI measurements received a head pedestal, similar to the one used to hold the goggle frame in the form deprivation group, to maintain position in the PCI. Using the Purkinje image as a reference, the PCI aiming laser was centered on the eye, and the measuring laser was directed into the eye. The reflected laser peaks were analyzed to determine the length of the eye from the anterior surface of the cornea to the retinal pigment epithelium/Bruch membrane interface [41].

Axial length was also measured using 1310 nm SD-OCT (Bioptigen, Durham, NC), as previously described [41]. Briefly, anesthetized mice were stabilized in a heated (36 °C) holder using a bite bar. A radial scan was performed first to aid in proper alignment in the center of pupil. A total of 100 scans were collected from five recordings (20 scans each) for each eye. Following the testing, mice were injected with yohimbine (2.1 mg/kg) to reverse the effects of xylazine to speed recovery and prevent corneal lesions [42].

Axial length was measured by placing calipers on the captured SD-OCT images using commercial software (Bioptigen), as previously described [41]. Axial length was measured from the anterior surface of the cornea to the retinal pigment epithelial/choroid interface. Some mice underwent PCI and SD-OCT measurements while others had only SD-OCT imaging. Since the PCI and SD-OCT measurements have good agreement [41], values from the instruments were averaged together to create an average value per eye.

### Retinal dopamine quantification

Steady-state levels of dopamine (DA) and DOPAC were measured using high-performance liquid chromatography with coulometric detection, as previously described [43]. Briefly, mice were euthanized by cervical dislocation 4–6 h into the light cycle and retinas frozen immediately on dry ice. Retinas were homogenized (0.1 N HClO_4_ containing 0.01% sodium metabisulfite and 50 ng/mL internal standard 3, 4 dihydroxybenzylamine hydrobromide) and centrifuged. The DA and DOPAC levels were determined in the supernatant fraction with high-performance liquid chromatography with coulometric detection. For analysis, the DA and DOPAC levels were compared between strains, combining ages from 4 to 12 weeks (WT, n=19; *rd1*, n=12; *rd10*, n=13; two to six retinas taken every 2 weeks). For the FD-treated animals, retinas were collected 48 h following the measurements at 2 weeks of goggling (WT: n=3 goggled, n=6 naïve control; *rd1*: n=14 goggled, n=11 naïve control; *rd10*: n=8 goggled, n=10 naïve control).

### Data analysis

All statistical analysis of the WT, *rd1*, and *rd10* mice in each experiment was performed with commercial software (SigmaStat 3.5, Aspire Software International, Ashburn, VA). For refractive error and axial length data on non-form deprivation eyes, the right eye and left eye measurements were averaged together to create a single value for each mouse. In the form deprivation group, the differences between the treated and untreated eyes were used to compare myopic shift across age. Axial length measurements were normalized to individual axial lengths at 4 weeks of age (baseline) to remove any effects of differences in body size on eye size [44-46] and the differences between the goggled and opposite eyes were calculated to determine axial shift. Comparisons between different strains and treatment groups were analyzed with repeated-measures analysis of variance (RM-ANOVA) or one-way ANOVA and Holm-Sidak post-hoc tests to determine significance in strains, age, and their interaction (p<0.05). The values at each time point are expressed as mean±standard error of the mean (SEM). Pearson’s correlation was performed between dopamine, DOPAC, and DOPAC/dopamine ratios versus myopic or axial shift in all three strains, plus the *nob* mice. The data from the *nob* mice were previously published [11] with supplementation of additional data for a total of n=33 for myopic shift, n=18 for axial length shift, and n=72 for dopamine data.

## Results

### Refractive development with normal visual input

#### Relative hyperopic refractions in rd1 and rd10 mice

**Figure 1:**
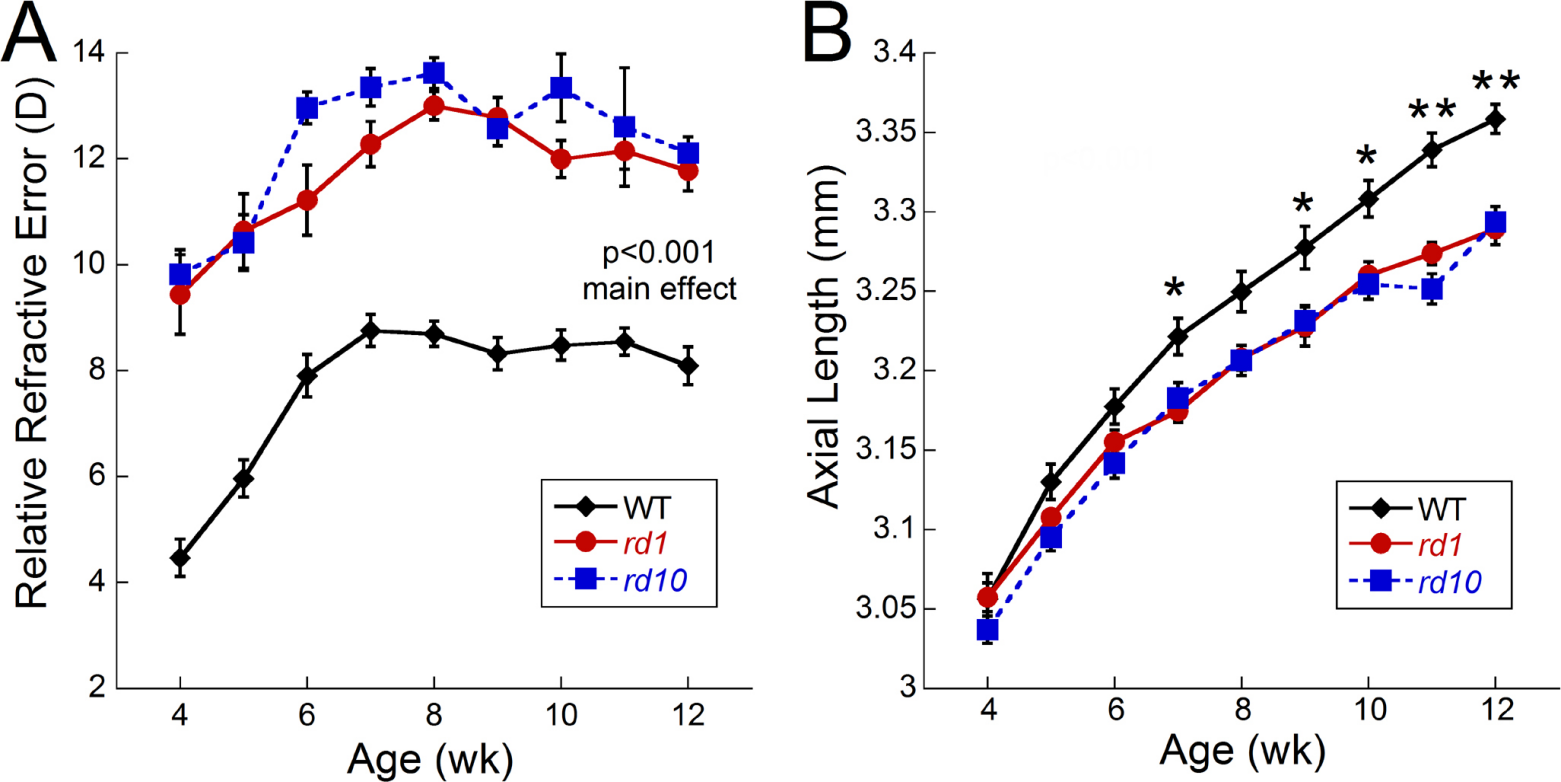
Refractive development of wild-type, *rd1*, and *rd10* mice. **A**: *Rd1* and *rd10* mice have more hyperopic refractions compared to wild-type (WT) control mice throughout the development period (repeated-measures analysis of variance [RM-ANOVA], main effect of treatment, F_(2,501)_=76.3, p<0.001). No statistical differences were found between *rd1* and *rd10* mice (WT, n=29; *rd1*, n=17; *rd10*, n=13). **B**: Axial length measurements were significantly shorter by 7 weeks of age in *rd1* and *rd10* mice compared to WT (RM-ANOVA F_(16, 252)_=6.95, p<0.001; WT, n=11; *rd1*, n=11; *rd10*, n=10). Data are expressed as mean±standard error of the mean (SEM). Post-hoc analysis: *p<0.02; **p<0.001.

From 4 to 12 weeks of age, the *rd1* and *rd10* mice had significantly more hyperopic refractions compared to the WT mice when raised in normal laboratory conditions (Figure 1A; RM- ANOVA, main effect of treatment, F_(2,501)_=76.3, p<0.001). However, no differences were detected between the *rd1* and *rd10* mice at any age. At 4 weeks of age, the WT mice had refractions of 4.47±0.35 D, while the *rd1* and *rd10* mice were approximately 5 diopter (D) more hyperopic (9.44±0.75 D and 9.82±0.46 D, respectively). All strains showed an increase in refractive error from 4 to 8 weeks of age with the WT mice shifting an average of 4.23 D (8.70±0.24 D at 8 weeks old) and the *rd1* and *rd10* mice shifting 3.6 and 3.8 D, respectively (*rd1* 13.00±0.27 D; *rd10* 13.62±0.29 D). From 8 to 12 weeks of age, refractions in the WT mice plateaued between 8 and 9 D (Figure 1A), while the *rd1* and *rd10* mice plateaued between 11 and 13 D (Figure 1A; 12 week refractions: *rd1*, 11.77±0.38 D; *rd10*, 12.11±0.30 D).

#### Shorter axial lengths in rd1 and rd10 mice

All three strains had similar axial lengths at 4 weeks of age followed by a slower rate of growth in the *rd1* and *rd10* mice that resulted in significantly longer eyes in the WT mice at 7 weeks (Figure 1B; RM-ANOVA F_(16, 252)_=6.95, p<0.001). At 4 weeks of age, the WT mice measured 3.06±0.02 mm, while the *rd1* and *rd10* mice had similar lengths of 3.06±0.01 and 3.04±0.03 mm, respectively. At 7 weeks, the WT mice had axial lengths of 3.22±0.01 mm, while the *rd1* and *rd10* mice had significantly shorter eyes (3.17±0.01 and 3.18±0.03 mm; post-hoc p=0.02). At 12 weeks, the WT eyes were significantly larger than the *rd1* and *rd10* eyes (Figure 1B; WT: 3.36±0.01mm, *rd1* 3.29±0.01, *rd10* 3.29±0.02mm; post-hoc p<0.002).

### Form deprivation visual conditions

#### Greater susceptibility to form deprivation myopia in rd1 and rd10 mice

**Figure 2:**
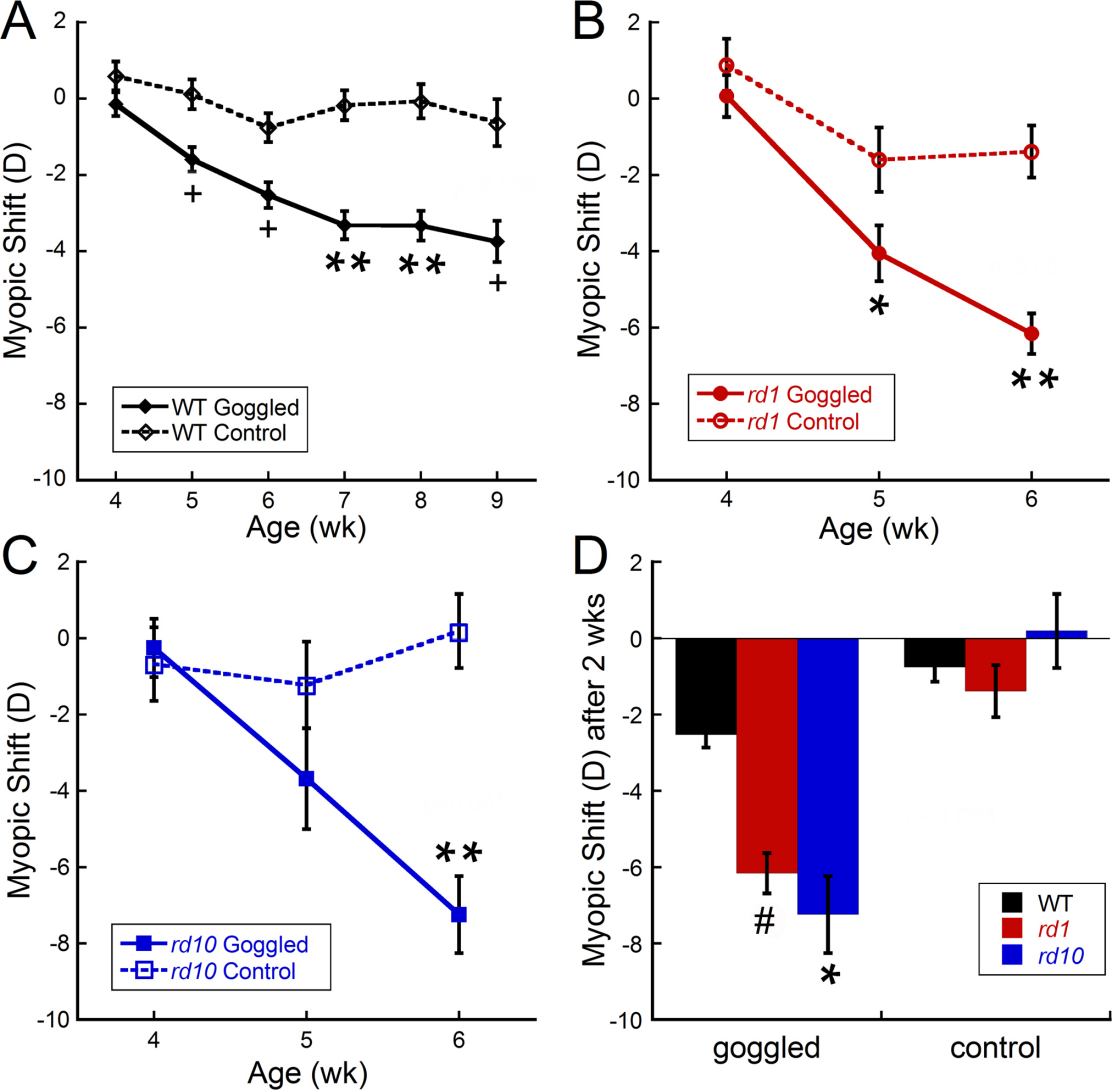
Refractive error changes (difference between right and left eyes) in wild-type, *rd1*, and *rd10* mice after form deprivation, starting at 4 weeks of age. **A**: Wild-type (WT) mice developed a significant myopic shift in goggled eyes by 1 week that steadily increased across the 5 weeks of form deprivation (FD; repeated-measures analysis of variance [RM-ANOVA] F_(5,253)_=3.49, p=0.005). **B**: *Rd1* mice developed a larger myopic shift than naïve controls after 1 week of goggling that further increased after 2 weeks of FD (RM-ANOVA, F_(2,111)_=6.97, p=0.002). **C**: *Rd10* mice developed a dramatic myopic shift that was significantly different from that of the controls after 2 weeks of goggling (RM-ANOVA, F_(2,81)_=9.12, p<0.001). **D**: After 2 weeks of goggling, the *rd1* and *rd10* mice had significantly greater myopic shifts than the WT mice (ANOVA, F_(2,118)_=8.69, p<0.001; WT: n=28 goggled, n=21 naïve controls; *rd1*: n=25 goggled, n=17 naïve controls; *rd10*: n=14 goggled, n=14 naïve controls). Symbols and bars represent average±standard error of the mean (SEM). Post-hoc analysis: ^#^p<0.05; *p<0.02; ^+^p<0.005; **p<0.001.

As previously reported [11], the WT mice showed a significant and progressive myopic shift with form deprivation (Figure 2A; RM-ANOVA F_(5,253)_=3.49, p=0.005). After 1 week of FD, the goggled eyes were 1.59±0.43 D more myopic than the contralateral eyes, which further decreased to 3.81±0.48 D after 5 weeks of FD. Age-matched naïve WT mice had only 0.25±0.17 D difference between eyes across the same treatment period.

In comparison, the *rd1* mice responded to FD with a significant myopic shift of −6.14±0.62 D by 2 weeks post goggling, compared to the untreated *rd1* controls, which showed a difference of −1.32±0.87 D between the eyes after 2 weeks (Figure 2B; RM-ANOVA, F_(2,111)_=6.97, p=0.002). *Rd10* mice showed a similar response to FD as the *rd1* mice. A significant myopic shift of −7.38±1.46 D was measured in the *rd10* mice with FD compared to a difference of 0.53±0.87 D between the eyes of the untreated control mice (Figure 2C; RM-ANOVA, F_(2,81)_=9.12, p<0.001).

The *rd1* and *rd10* mice had a significantly faster and more robust response to FD than the WT mice (Figure 2D; ANOVA, F_(2,118)_=8.69, p<0.001). Although a −6–7 D myopic shift was observed in the *rd1* and *rd10* mice after only 2 weeks of FD, the WT mice only shifted by < −3 D. No differences in refractive shifts were found in the FD response between the *rd1* and *rd10* mice or between eyes in the untreated control mice.

#### Axial length changes with form deprivation myopia

**Figure 3:**
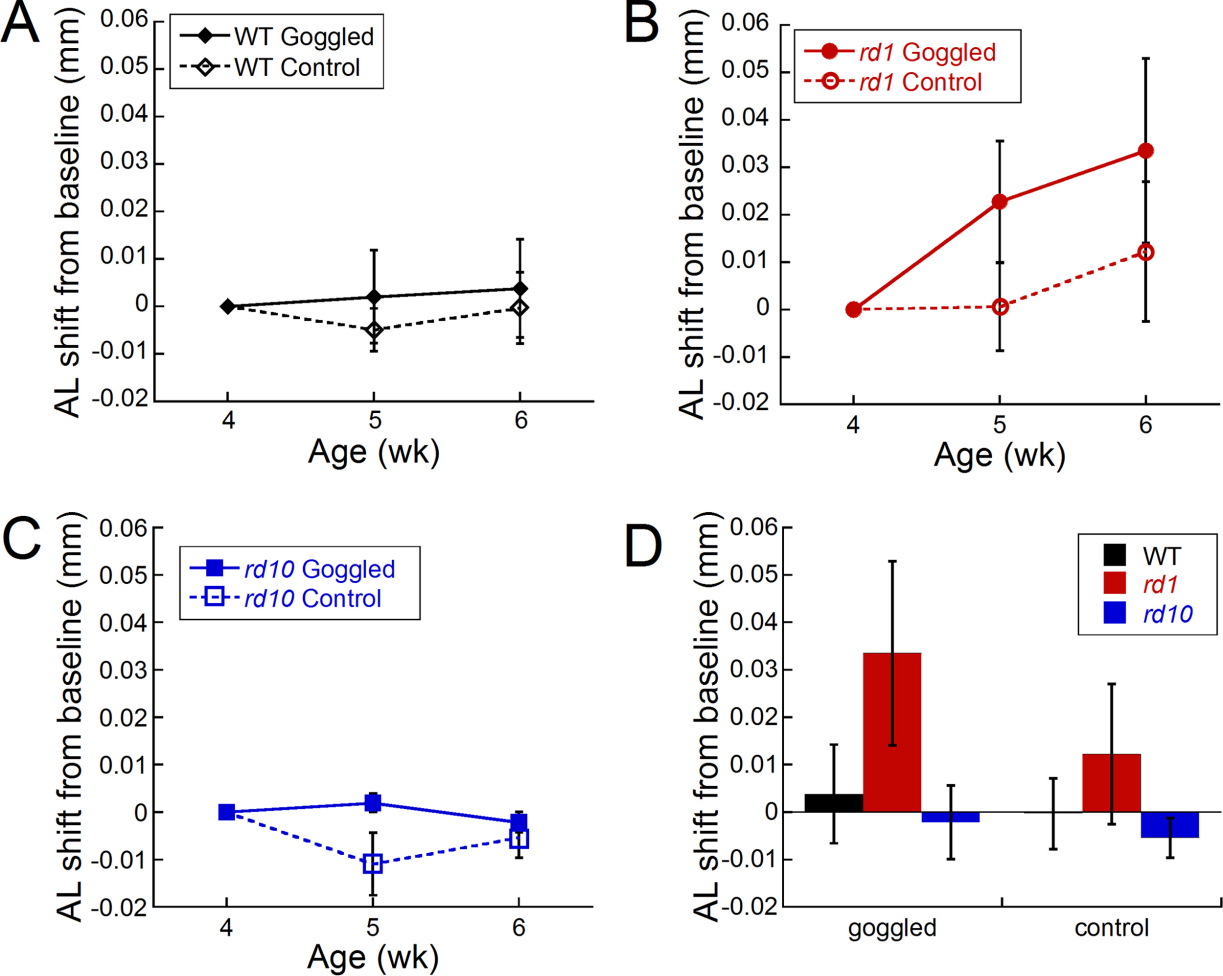
Axial length shifts (goggled minus opposite eyes) from baseline in wild-type, *rd1*, and *rd10* mice after form deprivation. **A**: Wild-type (WT) mice developed a weak trend for longer axial length shifts in goggled eyes compared to controls. **B**: *Rd1* mice showed a trend for longer axial length shifts after 1 week of goggling that further increased after 2 weeks of form deprivation (FD). **C**: The goggled eyes of *rd10* mice showed no differences compared to the naïve controls after 2 weeks of goggling. **D**: After 2 weeks of FD, the goggled WT and *rd1* eyes had slightly longer axial length shifts from baseline compared to the naïve control eyes (WT: n=17 goggled, n=13 naïve controls; *rd1*: n=13 goggled, n=11 naïve controls; *rd10*: n=14 goggled, n=14 naïve controls). Symbols and bars represent average±standard error of the mean (SEM).

After 2 weeks of form deprivation, the WT mice showed only a slight trend for greater axial shifts (difference between the two eyes normalized to baseline) in the goggled versus the control eyes (Figure 3A; 0.004±0.010 and 0.00±0.008 mm, respectively). With the same goggling treatment, the *rd1* mice had a larger, but non-significant trend for longer axial lengths shifts between the eyes from baseline after 1 and 2 weeks of goggling (Figure 3B; goggled axial shift from baseline: 0.034±0.013 mm; control axial shift from baseline 0.012±0.015 mm). The axial shifts in the *rd10* mice were slightly larger in the goggled versus control eyes after 1 week of FD (0.002±0.010 and −0.011±0.007 mm, respectively), but not at 2 weeks (Figure 3C; −0.002±0.008 and −0.005±0.004 mm, respectively). A comparison of the axial shift from baseline after 2 weeks of FD in the goggled mice of all three genotypes compared to control revealed a trend for all goggled eyes to have longer eyes than the corresponding controls (Figure 3D; WT: >1200%, *rd1*: >170%, *rd10*: >60% longer).

### Influence of dopamine on refractive development in *rd1* and *rd10* mice

#### DOPAC levels decreased in rd1 and rd10 mice

**Figure 4:**
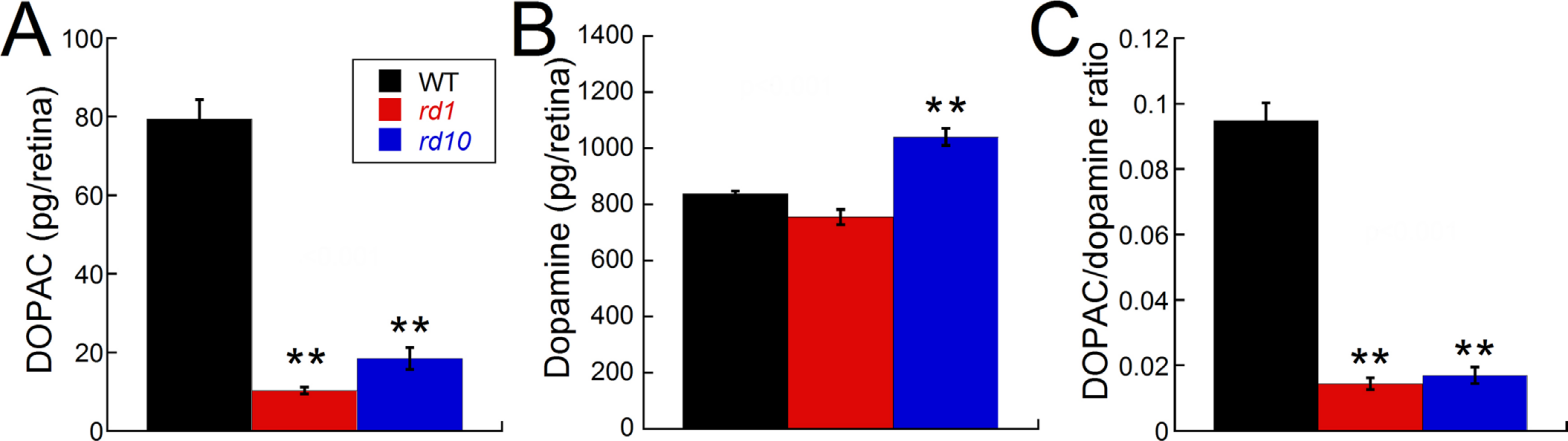
Dopamine and 3,4-dihydroxyphenylacetic acid levels in wild-type, *rd1*, and *rd10* retinas with normal visual experience. **A**: 3,4-dihydroxyphenylacetic acid (DOPAC) levels were significantly lower in the *rd1* and *rd10* retinas compared to the wild-type (WT) retinas (analysis of variance [ANOVA] F_(2,43)_=70.37, p<0.001). **B**: The dopamine levels were significantly higher in the *rd10* retinas compared to the WT or *rd1* retinas (ANOVA F_(2,43)_=11.72, p<0.001). **C**: The DOPAC/dopamine (DA) ratio was significantly lower in the *rd1* and *rd10* retinas compared to the WT retinas (F_(2,43)_=88.47, p<0.001; WT, n=19; *rd1*, n=12; *rd10*, n=13). Each bar represents the standard error of the mean (SEM). Post-hoc analysis: **p<0.001.

With normal visual input, retinal dopamine metabolism was significantly decreased in the *rd1* and *rd10* mice compared to the WT mice, as indicated by the low levels of DOPAC, the primary dopamine metabolite in the retina (Figure 4A; F_(2,43)_=70.37, p<0.001). In contrast, the dopamine levels were similar between the WT and *rd1* mice and significantly greater in the *rd10* mice (Figure 4B; F_(2,43)_=11.72, p<0.001). The DOPAC/dopamine ratio, an indicator of dopamine turnover, was also significantly decreased in the *rd1* and *rd10* mice compared to the WT mice (Figure 4C; F_(2,43)_=88.47, p<0.001).

#### Dopamine and myopia susceptibility in rd1 and rd10 mice

**Figure 5:**
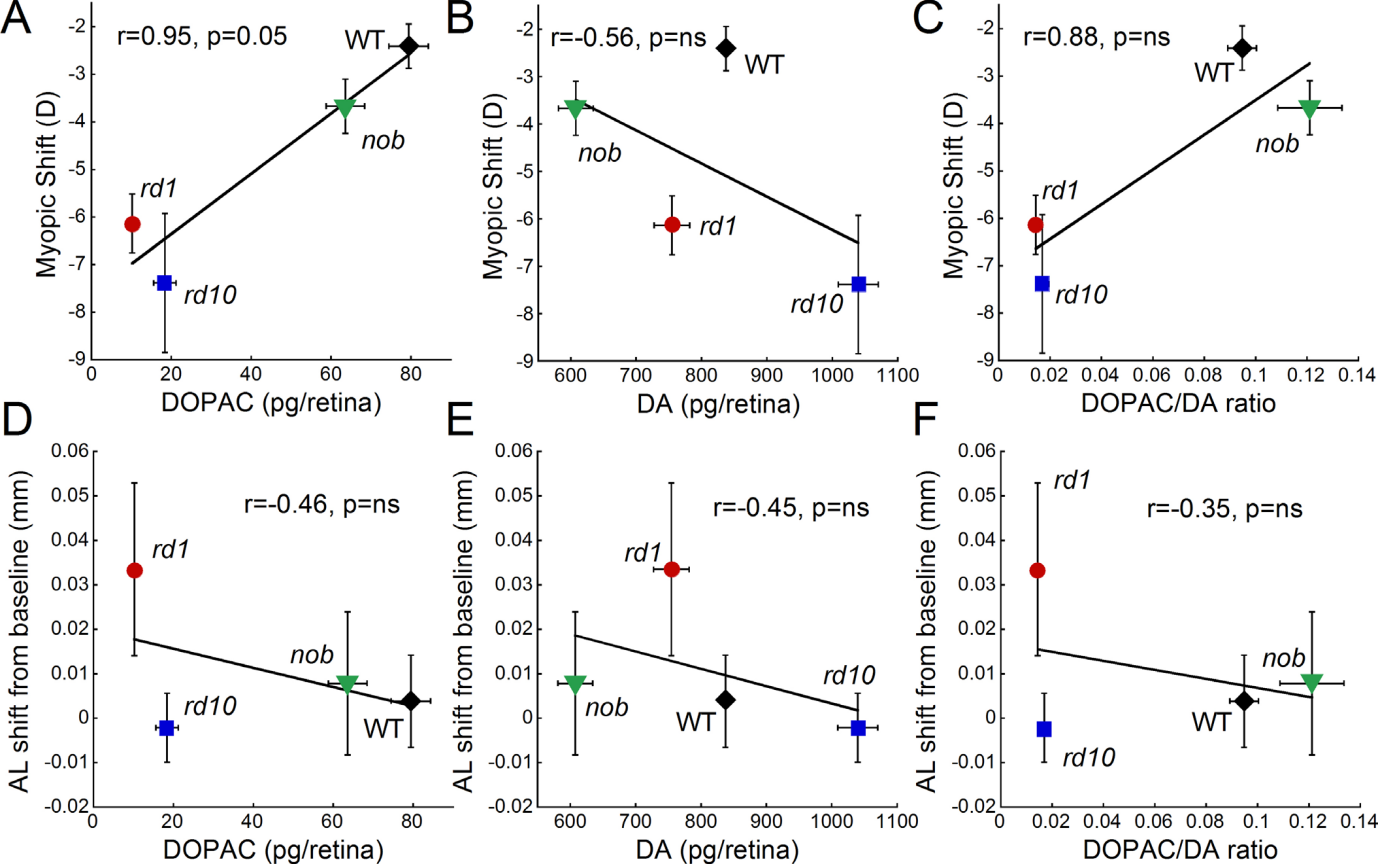
The influence of dopamine levels on form deprivation myopia susceptibility. The 3,4-dihydroxyphenylacetic acid (DOPAC) and dopamine (DA) levels and the DOPAC/DA ratio are the average levels obtained from retinas under normal visual conditions for each strain. These dopamine levels are plotted against the average myopic shift (**A**–**C**) or the axial shift from baseline (**D**–**F**) for each strain. Myopic shift was most strongly correlated with DOPAC levels across strains (Pearson’s correlation r=0.94, p=0.05), while basal state DA levels did not predict susceptibility to myopia (Pearson’s correlation r=–0.56). DA and DA metabolism (DOPAC levels) showed the strongest correlation with axial length shifts from baseline across strains (Pearson’s correlation r=–0.45 and r=–0.46, respectively), although DA turnover (DOPAC/DA ratio) had similar trends (Pearson’s correlation r=–0.35). Data for *rd1*, *rd10*, and wild-type (WT) retinas were obtained from this study, while the data from *nob* retinas were a combination of data obtained from [11] and recent additional measurements (n=33 for myopic shift, n=18 for axial length shift, and n=72 for dopamine data). Symbols and bars represent average±standard error of the mean (SEM).

After 2 weeks of form deprivation, the DOPAC and dopamine levels and the DOPAC/dopamine ratios did not significantly change between the goggled and opposite or control eyes in the *rd1*, *rd10*, or WT mice (data not shown). To determine if the endogenous levels of dopamine and DOPAC in the different genotypes influenced myopia susceptibility, we examined the correlation between DOPAC, dopamine, and the DOPAC/dopamine ratio under normal visual conditions with myopic and axial shifts after FD. In addition, to analyze the myopic shift and dopamine levels, we included data on the *nob* mice, which also have altered dopamine levels and increased susceptibility to myopia [11]. Figure 5 indicates that decreased dopamine metabolism (DOPAC levels) correlated with greater myopic shifts at nearly significant levels (Pearson’s correlation r=0.95, p=0.05). Dopamine turnover (DOPAC/DA ratio) also correlated with greater myopic shifts (Pearson’s correlation r=0.88, p=ns), while dopamine was not as closely correlated (Pearson’s correlation r= –0.56, r=ns). However, decreased DOPAC, dopamine, and DOPAC/DA ratios were weakly associated with increased axial length shifts from baseline (Pearson’s correlation, r= –0.46, r= –0.45, r= –0.35, respectively; p=ns).

## Discussion

The *Pde6b* mutation results in altered refractive development in *rd1* and *rd10* mice. Under laboratory conditions, refractive errors were more hyperopic and axial lengths shorter in the *rd1* and *rd10* mice compared to the WT mice. However, the *rd1* and *rd10* mice were more susceptible to form deprivation myopia than the WT mice, developing >–3 D myopic shift in 1 week and >–6D in 2 weeks (Figure 2). Interestingly, the mice did not show any significant corresponding axial shifts with form deprivation (Figure 3). Since dopamine has been postulated as an important retinal signal in regulating eye growth, we hypothesize that these changes could be due to alterations in dopamine metabolism in these retinal degeneration models.

Dopamine activity has been reported to be disrupted in several models of retinal degeneration. Although dopaminergic amacrine cells are morphologically intact [47,48], dopamine turnover and light-induced increases in dopamine synthesis are absent [35]. The Royal College of Surgeons (RCS) rat is a model of retinitis pigmentosa with a retinal pigment epithelium defect that begins photoreceptor degeneration at about 12 days of age [49]. RCS rats have increased dopamine and DOPAC levels in response to light onset that is similar to non-dystrophic rats until post-natal day 30 when dopamine and its metabolite become significantly lower [50,51]. Furthermore, treatment with a D1 antagonist failed to elicit a response from horizontal cells in RCS rats, perhaps due to the lower level of dopamine in these retinas creating a floor effect [36]. In *rds−/−* mice, a model of photoreceptor degeneration due to a peripherin mutation, dopamine synthesis and turnover are also low compared to WT, and the typical increase in dopamine with light onset is reduced, as well [34]. In the *rd1* retina, dopaminergic neurons have less spontaneous bursting activity, and only a small population of neurons remain light-sensitive [48]. Many of these reported abnormalities in dopamine activity in retinal dystrophy models occur before photoreceptor degeneration is complete, suggesting early abnormalities in dopaminergic pathways in retinal disease [34,36] that may influence retinal function and refractive development in later periods of life. Thus, early dysfunction in dopamine neurons in the *rd1* and *rd10* mice may establish a retinal environment that predisposes the retina to abnormal refractive development. Under normal visual conditions, the low DOPAC levels, but normal or increased dopamine levels in *rd1* and *rd10* mice, respectively, indicate that dopamine is synthesized, but not metabolized at the same rate as in controls, potentially indicating low dopamine release. Thus, the low level of dopamine metabolism in the *rd1* and *rd10* retinas at the time of goggling (4 weeks of age) may accelerate the myopic response to FD, as suggested by strains with the lowest DOPAC levels having the greatest myopic shifts with form deprivation (Figure 5A). The potential link between dopamine metabolism and myopia susceptibility is further supported by data from the *nob* mice, which lack ON pathway transmission and do not undergo photoreceptor degeneration [11,52,53]. Thus, this effect is not unique to eyes undergoing retinal degeneration.

Axial length is the ocular component most highly correlated with myopic refractions [3-5]. In this study, we normalized axial length measurements after form deprivation by examining the difference from baseline. We believe this approach allowed for a direct comparison of axial length shifts between genotypes in response to form deprivation because 1) body size is highly correlated with eye size [44-46,54], 2) murine body size can vary greatly by strain [55], and 3) axial ocular component dimensions are highly heritable [56]. Using this analysis method, axial length shifts showed a trend for larger axial length shifts in the *rd1* mice compared to the WT and *rd10* mice after 2 weeks of goggling. These axial length shifts corresponded to the predicted values based on the measured myopic shift when using the paraxial schematic eye calculations that a 5 micron change in axial length is equivalent to a 1 diopter change in refractive error [39,57]. In the WT mice, the −2.41±0.47 D refractive shift corresponded to a 4±10 µm axial length shift from the baseline, and in the *rd1* mice, a −6.14±0.62 D myopic shift corresponded to a 33±19 µm axial length shift from baseline (Figure 2 and Figure 3). However, the *rd10* mice developed the largest refractive shift (−7.38±1.46 D) and yet showed no axial length shifts from baseline (−2±8 µm; Figure 2 and Figure 3). This could be due to alterations in other parameters, such as corneal curvature or lens thickness, which may also explain similar axial lengths observed between the WT and *rd* strains with normal development. Another consideration is that the dopamine levels in the *rd10* mice were higher than the WT and *rd1* mice under normal conditions. If dopamine is a regulator of axial length, then perhaps this increase in the “stop” signal reduced the effects of form deprivation.

Although we have focused on the potential role of dopamine activity in these models for regulating eye growth, a reduced or absent number of photoreceptors in the *rd1* and *rd10* mice may also affect refractive development. With the severe degeneration in the *rd1* model, all rod and only a few cone photoreceptors would be present to create a visual signal during the critical period of refractive development; while in the *rd10* model, about 89% of cones would still be viable at P26 [26]. However, this difference in the rate of degeneration and the stage at which goggling occurred did not alter refractive development or the response to form deprivation between the *rd1* and *rd10* mice, suggesting that there may be a prerequisite number of photoreceptors necessary for the response or that another mechanism, such as defects in dopamine metabolism that control refractive development under normal and form deprivation conditions, is responsible. In addition, the similar phenotype observed between the *rd1* and *rd10* mice may indicate that retinal signaling pathway(s) have greater influence on eye growth than actual morphology. Comparisons with other retinal degenerative models that are slower and that have different mutations may provide further insight into the potential role of the photoreceptors in visually driven eye growth.

Retinal degeneration also alters several other growth factors and neurotransmitters that have been implicated in refractive eye growth signaling. For instance, multiple genes are upregulated during the early phases of photoreceptor apoptosis in the *rd10* mice, including fibroblast growth factor 2 [58]. Fibroblast growth factor 2 has been implicated as a regulator of ocular eye growth through scleral remodeling [59-61] and has been shown to have increased expression in form deprivation guinea pigs [62]. Additionally, Ɣ-aminobutyric acid (GABA)/melanopsin signaling may be enhanced in retinal degeneration, as shown in *rd1* mice [48], and GABA may stimulate eye growth [63-65].

Remodeling of the inner retinal neurons after the death of photoreceptors has been well established in models of retinal degeneration [27,31,32,66,67]. This remodeling alters normal visual circuitry and potentially influences eye growth signaling. For example, while cell bodies of inner retinal cells remain in *rd1* retinas after rod and cone death at P30, the axonal complexes of rod bipolar cells and horizontal cells are disorganized, and the dendrites are retracted [27]. Similar changes have been reported in the *rd10* mice by P40 [31,32,68,69]. However, other aspects, such as the stratification of the ON and OFF bipolar cell terminals, remain intact in *rd10* mice until 9.5 months of age [69]. In some retinal degeneration models, cholinergic amacrine cells have been shown to undergo synaptic remodeling in late stage disease [70], and cholinergic receptors have been implicated in myopia [6,71,72]. Much of this synaptic remodeling occurs between 30 and 90 days of age and beyond, a critical window for refractive development. Since the signaling pathway(s) underlying refractive development have not been firmly established, it is unknown what alterations in the degenerating retina are most disruptive to eye growth signaling and the timing of these alterations within the critical period for emmetropization.

Furthermore, in Figure 5, the *rd1* and *rd10* mice have larger myopic shifts with FD than the *nob* mice. This might imply that defects in the phototransduction cascade cause more severe myopia and abnormal dopamine activity than a mutation in a second-order neuron protein. Thus, the ability of photoreceptors to respond correctly to light may be more important to signaling eye growth than retinal defects in higher-order signaling components. An evaluation of eye growth and susceptibility of myopia in additional strains with photoreceptor and inner retinal defects is needed to further explore this possibility.

In conclusion, this study indicates that retinal degeneration in the *rd1* and *rd10* mice increased susceptibility to form deprivation myopia, while producing relatively hyperopic refractions under normal visual conditions. Our data support the hypothesis that dopamine activity may act as a “stop signal” for axial eye growth and provides some evidence that basal levels of dopamine activity in the retina may influence the response to visual disruptions, such as form deprivation. The clinical relevance of these results is that retinal degeneration alone may not produce myopia, but provides a risk factor that increases myopia susceptibility when combined with myopigenic visual conditions. Future studies with other mouse models with dopamine defects will provide further evidence of these potential roles of dopamine in refractive development.

## References

[1] Vitale S, Sperduto RD, Ferris FL (2009). Increased prevalence of myopia in the United States between 1971–1972 and 1999–2004.. Arch Ophthalmol.

[2] Jung SK, Lee JH, Kakizaki H, Jee D (2012). Prevalence of myopia and its association with body stature and educational level in 19-year-old male conscripts in seoul, South Korea.. Invest Ophthalmol Vis Sci.

[3] Wallman J, Winawer J (2004). Homeostasis of eye growth and the question of myopia.. Neuron.

[4] Nickla DL Ocular diurnal rhythms and eye growth regulation: Where we are 50 years after Lauber. Exp Eye Res 2013114:25-34 23298452 10.1016/j.exer.2012.12.013 PMC3742730

[5] Flitcroft DI Is myopia a failure of homeostasis? Exp Eye Res 2013114:16-24 23454097 10.1016/j.exer.2013.02.008

[6] Stone RA Pardue MT Iuvone PM Khurana TS Pharmacology of myopia and potential role for intrinsic retinal circadian rhythms. Exp Eye Res 2013114:35-47 23313151 10.1016/j.exer.2013.01.001 PMC3636148

[7] Feldkämper M, Schaeffel F (2003). Interactions of genes and environment in myopia.. Dev Ophthalmol.

[8] Saw SM, Gazzard G, Shih-Yen EC, Chua WH (2005). Myopia and associated pathological complications.. Ophthalmic Physiol Opt.

[9] Laties A, Stone R. Ametropia in retinal disorders, In: Anderson RE, Hollyfield JG, and LaVail MM, Editors. Retinal Degenerations. Boca Raton: CRC Press; 1991. p. 383–390.

[10] Miyake Y, Yagasaki K, Horiguchi M, Kawase Y, Kanda T (1986). Congenital stationary night blindness with negative electroretinogram. A new classification.. Arch Ophthalmol.

[11] Pardue MT, Faulkner AE, Fernandes A, Yin H, Schaeffel F, Williams RW, Pozdeyev N, Iuvone PM (2008). High susceptibility to experimental myopia in a mouse model with a retinal on pathway defect.. Invest Ophthalmol Vis Sci.

[12] Chui TY, Bissig D, Berkowitz BA, Akula JD (2012). Refractive Development in the "ROP Rat".. J Ophthalmol.

[13] Lavanya R, Kawasaki R, Tay WT, Cheung GC, Mitchell P, Saw SM, Aung T, Wong TY (2010). Hyperopic refractive error and shorter axial length are associated with age-related macular degeneration: the Singapore Malay Eye Study.. Invest Ophthalmol Vis Sci.

[14] Age-Related Eye Disease Study Research G (2000). Risk factors associated with age-related macular degeneration. A case-control study in the age-related eye disease study: Age-Related Eye Disease Study Report Number 3.. Ophthalmology.

[15] Jonas JB, Nangia V, Kulkarni M, Gupta R, Khare A (2012). Associations of early age-related macular degeneration with ocular and general parameters. The Central India Eyes and Medical Study.. Acta Ophthalmol.

[16] Smith M, Whittock N, Searle A, Croft M, Brewer C, Cole M (2007). Phenotype of autosomal dominant cone-rod dystrophy due to the R838C mutation of the GUCY2D gene encoding retinal guanylate cyclase-1.. Eye (Lond).

[17] Pras E, Abu A, Rotenstreich Y, Avni I, Reish O, Morad Y, Reznik-Wolf H, Pras E (2009). Cone-rod dystrophy and a frameshift mutation in the PROM1 gene.. Mol Vis.

[18] Sieving PA, Fishman GA (1978). Refractive errors of retinitis pigmentosa patients.. Br J Ophthalmol.

[19] McLaughlin ME, Ehrhart TL, Berson EL, Dryja TP (1995). Mutation spectrum of the gene encoding the beta subunit of rod phosphodiesterase among patients with autosomal recessive retinitis pigmentosa.. Proc Natl Acad Sci USA.

[20] McLaughlin ME, Sandberg MA, Berson EL, Dryja TP (1993). Recessive mutations in the gene encoding the beta-subunit of rod phosphodiesterase in patients with retinitis pigmentosa.. Nat Genet.

[21] Bowes C, Li T, Danciger M, Baxter LC, Applebury ML, Farber DB (1990). Retinal degeneration in the rd mouse is caused by a defect in the beta subunit of rod cGMP-phosphodiesterase.. Nature.

[22] Pittler SJ, Baehr W (1991). Identification of a nonsense mutation in the rod photoreceptor cGMP phosphodiesterase beta-subunit gene of the rd mouse.. Proc Natl Acad Sci USA.

[23] Chang B, Hawes NL, Pardue MT, German AM, Hurd RE, Davisson MT, Nusinowitz S, Rengarajan K, Boyd AP, Sidney SS, Phillips MJ, Stewart RE, Chaudhury R, Nickerson JM, Heckenlively JR, Boatright JH (2007). Two mouse retinal degenerations caused by missense mutations in the beta-subunit of rod cGMP phosphodiesterase gene.. Vision Res.

[24] Pittler SJ, Keeler CE, Sidman RL, Baehr W (1993). PCR analysis of DNA from 70-year-old sections of rodless retina demonstrates identity with the mouse rd defect.. Proc Natl Acad Sci USA.

[25] Keeler CE (1924). The inheritance of a retinal abnormality in white mice.. Proc Natl Acad Sci USA.

[26] Carter-Dawson LD, LaVail MM, Sidman RL (1978). Differential effect of the rd mutation on rods and cones in the mouse retina.. Invest Ophthalmol Vis Sci.

[27] Strettoi E, Porciatti V, Falsini B, Pignatelli V, Rossi C (2002). Morphological and functional abnormalities in the inner retina of the rd/rd mouse.. J Neurosci.

[28] LaVail MM, Matthes MT, Yasumura D, Steinberg RH (1997). Variability in rate of cone degeneration in the retinal degeneration (rd/rd) mouse.. Exp Eye Res.

[29] Jiménez AJ, Garcia-Fernandez JM, Gonzalez B, Foster RG (1996). The spatio-temporal pattern of photoreceptor degeneration in the aged rd/rd mouse retina.. Cell Tissue Res.

[30] Chang B, Hawes NL, Hurd RE, Davisson MT, Nusinowitz S, Heckenlively JR (2002). Retinal degeneration mutants in the mouse.. Vision Res.

[31] Gargini C, Terzibasi E, Mazzoni F, Strettoi E (2007). Retinal organization in the retinal degeneration 10 (rd10) mutant mouse: a morphological and ERG study.. J Comp Neurol.

[32] Barhoum R, Martinez-Navarrete G, Corrochano S, Germain F, Fernandez-Sanchez L, de la Rosa EJ, de la Villa P, Cuenca N (2008). Functional and structural modifications during retinal degeneration in the rd10 mouse.. Neuroscience.

[33] Stone RA, Lin T, Laties AM, Iuvone PM (1989). Retinal dopamine and form-deprivation myopia.. Proc Natl Acad Sci USA.

[34] Nir I, Iuvone PM (1994). Alterations in light-evoked dopamine metabolism in dystrophic retinas of mutant rds mice.. Brain Res.

[35] Djamgoz MB, Hankins MW, Hirano J, Archer SN (1997). Neurobiology of retinal dopamine in relation to degenerative states of the tissue.. Vision Res.

[36] Hankins M, Ikeda H (1994). Early abnormalities of retinal dopamine pathways in rats with hereditary retinal dystrophy.. Doc Ophthalmol.

[37] Doyle SE, McIvor WE, Menaker M (2002). Circadian rhythmicity in dopamine content of mammalian retina: role of the photoreceptors.. J Neurochem.

[38] Faulkner AE, Kim KK, Iuvone PM, Pardue MT (2007). Head-mounted goggles for murine form deprivation myopia.. J Neurosci Methods.

[39] Schaeffel F, Burkhardt E, Howland HC, Williams RW (2004). Measurement of refractive state and deprivation myopia in two strains of mice.. Optom Vis Sci.

[40] Schaeffel F (2008). Test systems for measuring ocular parameters and visual function in mice.. Front Biosci.

[41] Park H, Qazi Y, Tan C, Jabbar SB, Cao Y, Schmid G, Pardue MT (2012). Assessment of axial length measurements in mouse eyes.. Optom Vis Sci.

[42] Turner PV, Albassam MA (2005). Susceptibility of rats to corneal lesions after injectable anesthesia.. Comp Med.

[43] Nir I, Haque R, Iuvone PM (2000). Diurnal metabolism of dopamine in the mouse retina.. Brain Res.

[44] Wang D, Ding X, Liu B, Zhang J, He M (2011). Longitudinal changes of axial length and height are associated and concomitant in children.. Invest Ophthalmol Vis Sci.

[45] Yin G Wang YX Zheng ZY Yang H Xu L Jonas JBand Beijing Eye Study G, Ocular axial length and its associations in Chinese: the Beijing Eye StudyPLoS ONE 2012 7 e43172 22927949 10.1371/journal.pone.0043172 PMC3424225

[46] Prashar A, Hocking PM, Erichsen JT, Fan Q, Saw SM, Guggenheim JA (2009). Common determinants of body size and eye size in chickens from an advanced intercross line.. Exp Eye Res.

[47] Sharma RK (2001). Development and survival of tyrosine hydroxylase containing neurons in RCS rat retinae.. Curr Eye Res.

[48] Atkinson CL, Feng J, Zhang DQ (2013). Functional integrity and modification of retinal dopaminergic neurons in the rd1 mutant mouse: roles of melanopsin and GABA.. J Neurophysiol.

[49] LaVail MM, Battelle BA (1975). Influence of eye pigmentation and light deprivation on inherited retinal dystrophy in the rat.. Exp Eye Res.

[50] Frucht Y, Melamed E (1984). The dopaminergic amacrine system and its response to light stimulation in rats with inherited retinal dystrophy.. Exp Eye Res.

[51] Vugler AA, Redgrave P, Hewson-Stoate NJ, Greenwood J, Coffey PJ (2007). Constant illumination causes spatially discrete dopamine depletion in the normal and degenerate retina.. J Chem Neuroanat.

[52] Gregg RG, Mukhopadhyay S, Candille SI, Ball SL, Pardue MT, McCall MA, Peachey NS (2003). Identification of the gene and the mutation responsible for the mouse nob phenotype.. Invest Ophthalmol Vis Sci.

[53] Pardue MT, McCall MA, LaVail MM, Gregg RG, Peachey NS (1998). A naturally occurring mouse model of X-linked congenital stationary night blindness.. Invest Ophthalmol Vis Sci.

[54] Larsen JS (1979). Axial length of the emmetropic eye and its relation to the head size.. Acta Ophthalmol (Copenh).

[55] Reed DR, Lawler MP, Tordoff MG (2008). Reduced body weight is a common effect of gene knockout in mice.. BMC Genet.

[56] Wang L, Povazay B, Chen YP, Hofer B, Drexler W, Guggenheim JA (2011). Heritability of ocular component dimensions in mice phenotyped using depth-enhanced swept source optical coherence tomography.. Exp Eye Res.

[57] Schmucker C, Schaeffel F (2004). In vivo biometry in the mouse eye with low coherence interferometry.. Vision Res.

[58] Samardzija M, Wariwoda H, Imsand C, Huber P, Heynen SR, Gubler A, Grimm C (2012). Activation of survival pathways in the degenerating retina of rd10 mice.. Exp Eye Res.

[59] Ritchey ER, Zelinka CP, Tang J, Liu J, Fischer AJ (2012). The combination of IGF1 and FGF2 and the induction of excessive ocular growth and extreme myopia.. Exp Eye Res.

[60] Gentle A, McBrien NA (2002). Retinoscleral control of scleral remodelling in refractive development: a role for endogenous FGF-2?. Cytokine.

[61] Rohrer B, Stell WK (1994). Basic fibroblast growth factor (bFGF) and transforming growth factor beta (TGF-beta) act as stop and go signals to modulate postnatal ocular growth in the chick.. Exp Eye Res.

[62] An J, Hsi E, Zhou X, Tao Y, Juo SH, Liang CL (2012). The FGF2 gene in a myopia animal model and human subjects.. Mol Vis.

[63] Schmid KL, Strasberg G, Rayner CL, Hartfield PJ (2013). The effects and interactions of GABAergic and dopaminergic agents in the prevention of form deprivation myopia by brief periods of normal vision.. Exp Eye Res.

[64] Stone RA, Liu J, Sugimoto R, Capehart C, Zhu X, Pendrak K (2003). GABA, experimental myopia, and ocular growth in chick.. Invest Ophthalmol Vis Sci.

[65] Chebib M, Hinton T, Schmid KL, Brinkworth D, Qian H, Matos S, Kim HL, Abdel-Halim H, Kumar RJ, Johnston GA, Hanrahan JR (2009). Novel, potent, and selective GABAC antagonists inhibit myopia development and facilitate learning and memory.. J Pharmacol Exp Ther.

[66] Lin B, Masland RH, Strettoi E (2009). Remodeling of cone photoreceptor cells after rod degeneration in rd mice.. Exp Eye Res.

[67] Strettoi E, Pignatelli V (2000). Modifications of retinal neurons in a mouse model of retinitis pigmentosa.. Proc Natl Acad Sci USA.

[68] Puthussery T, Gayet-Primo J, Pandey S, Duvoisin RM, Taylor WR (2009). Differential loss and preservation of glutamate receptor function in bipolar cells in the rd10 mouse model of retinitis pigmentosa.. Eur J Neurosci.

[69] Phillips MJ, Otteson DC, Sherry DM (2010). Progression of neuronal and synaptic remodeling in the rd10 mouse model of retinitis pigmentosa.. J Comp Neurol.

[70] Kuny S, Gaillard F, Mema SC, Freund PR, Zhang K, Macdonald IM, Sparrow JR, Sauve Y (2010). Inner retina remodeling in a mouse model of stargardt-like macular dystrophy (STGD3).. Invest Ophthalmol Vis Sci.

[71] Barathi VA Kwan JL Tan QS Weon SR Seet LF Goh LK Vithana EN Beuerman RW Muscarinic cholinergic receptor (M2) plays a crucial role in the development of myopia in mice. Dis Model Mech 20136:1146-1158 23649821 10.1242/dmm.010967 PMC3759334

[72] Ashby R, McCarthy CS, Maleszka R, Megaw P, Morgan IG (2007). A muscarinic cholinergic antagonist and a dopamine agonist rapidly increase ZENK mRNA expression in the form-deprived chicken retina.. Exp Eye Res.

